# On Infantile Convulsions

**Published:** 1859-01

**Authors:** George J. Ziegler

**Affiliations:** Philadelphia


*On Infantile Convulsions.* By GEORGE J. ZIEGLER, M. D., Philadelphia.

In the late discussion before the County Medical Society, on infantile convulsions, one of the speakers is reported to have said, that in those cases in which "we do not dare to bleed, he was often puzzled what to do." Now, as depletion and all other spoliative measures are being justly regarded with distrust, as not only fraught with much evil, but of doubtful benefit, especially in infantile affections, it is important to settle down upon some more certain and philosophic method of reducing arterial and nervous excitement, and of subduing their concomitants. As the experience and suggestions of every one are more or less useful in determining the proper course, I present the following.

In those disturbances of the vascular and nervous systems, attended with local hyperæmia and irregular or undue innervation of any particular part of the economy, and especially of the cerebro-spinal axis, followed by convulsions, I have found the following course of procedure most useful, in allaying the excitement, and in restoring the general vital equilibrium, without leaving any bad effects, or inflicting permanent injury, as bleeding and other perturbative measures so frequently do.

When the convulsions are connected with vascular engorgement of the cerebro-spinal system, as indicated by the congested eye, flushed face, and, in infants, by the fuller distended fontanels, and other well known symptoms, besides the enemata, sinapisms to the extremities, or mustard pediluviæ, etc., I apply sinapisms or other counter-irritants to the whole surface of the spine, from the occiput to the sacrum, and administer aconite internally. For children, I prefer the tincture of the leaves, as it can be given more freely, with less danger of over-drugging. It should be administered in small and somewhat frequent doses, according to the age of the patient, and necessity of the case. Thus, one drop every hour, or more or less frequently, according to circumstances, to a child one year old. The effect of this remedy, in allaying nervous excitement, reducing the active action of the heart and vascular system, in resolving congestion, and in subduing spasmodic action, is sometimes strikingly shown in the speedy disappearance of the blood from the eye, the calming down of the general system, and the subsidence of the spasms.

The veratrum viride is also another very powerful arterial sedative, and anti-spasmodic, applicable to these cases, and no doubt would often prove useful, either alone, or where smaller doses were preferred in combination with the aconite.

When the case is severe or very urgent, and even the small doses of these remedies cannot be so readily introduced into the system, or act with sufficient promptitude, the use of chloroform by inhalation, would be indicated.

Another apparently valuable method of resolving these and other congestions of the head and spine, as well as other parts of the trunk, is by the process termed *hæmostasia*, which is by ligating the limbs, and thus retaining the blood within them, and reducing the quantity of that fluid in the body proper. Hence, acting as a diverticulum, in withdrawing it from the affected organs.

Still another, and what promises to be a much more efficient plan, is by the method called *hæmospasia*, which acts on the principle of "dry cupping on a large scale," in withdrawing the blood to the surface and extremities. But the difficulty with this is that it cannot be so readily resorted to in private practice. It is, however, only noticed here to direct more attention to it, in the hope that it may be rendered more practical.

In those cases depending more especially upon cerebro-spinal congestion, cold applied to the spine, by means of ice and other agents, promises to be of much value, as it is a standard remedy in cerebral congestions and inflammations. Besides the evidence of experience itself is in its favor, as it has been successfully employed, not only in infantile spasm, but in tetanus, that most intractable of all convulsive affections.

In those cases in which the convulsion appears to be more exclusively connected with sympathetic irritation, or undue excitement of the general nervous system, hop poultices or other anodynes and antispasmodics, applied the whole length of the spine, in conjunction with the internal use of aconite or morphia, or the inhalation of chloroform, will usually soon quiet the system, and check the spasm.

The susceptibility to nervous excitement or convulsive action is, however, often more or less conneeted with general adynamia; and in these cases as well as in all others of irregular innervation from general debility, applications down the spine, of the stimulant anodynes and anti-spasmodics, with the judicious internal administration of the same, will generally have the desired effect. In this variety, the inhalation of ether, or of ether and chloroform combined, might sometimes be resorted to with advantage. Here, however, an after effort should always be made to overcome the predisposition, by a general tonic course of treatment.

Where the tendency to spasm is very active, and persist for any length of time, permanent counter irritation down the spine, in conjunction with appropriate internal medication, will be indicated.

As a general thing, then, the spasms dependent upon vascular and nervous excitement, with congestion of the cerebro spinal axis, counter-irritation by sinapisms, dry cups, or other means, to the spine, or the application of cold thereto, with the internal administration of aconite, or veratrum, or the inhalation of chloroform, will be most applicable. In addition to these, the treatment by the hæmostatic or hæmospastic process may also be put into practice.

In those cases in which nervous excitement predominates, the hop poultice, or other anodyne application tothe spine, with the internal exhibition of aconite, morphia, or chloroform by inhalation, will be more strongly indicated, presupposing, of course, in the spymathetic irritation, the removal of the offending cause.

In those cases connected with exhaustion or debility, the application to the spine of hops and garlic, or other anti-spasmodics, either separately or combined, with appropriate internal remedies, will be better adapted to fulfil the indications.

In fact, in all cases attention should be particularly directed to regulate and harmonize the circulation and innervation, and to tranquilize the general system, by suitable external and internal medication, with the non-spoliative, arterial and nervous sedatives and anti-spasmodics, according to the character, stage, type, intensity, complications, etc., of the derangement.

This plan of influencing the nervous, arterial, and general system, by medicating the spine, is of extensive application, and will often be followed by very beneficial effects. In one case of low fever, in which the patient was *in extremis,* the scale appeared to be turned in favor of recovery by the efficient aid obtained from the free use of spice plasters to the spine.

These very general and desultory remarks on infantile convulsions, are not designed to cover the whole ground, but merely invite attention to some few points not noticed in the recent discussion of that subject.—*Medical & Surgical Reporter.*

